# Editorial: Methods in Metabolomics 2022

**DOI:** 10.3389/fmolb.2023.1168941

**Published:** 2023-03-10

**Authors:** Joerg Buescher, Luciana Hannibal

**Affiliations:** ^1^ Max Planck Institute of Immunobiology and Epigenetics, Freiburg im Breisgau, Germany; ^2^ Laboratory of Clinical Biochemistry and Metabolism, Department of General Pediatrics, Adolescent Medicine and Neonatology, Faculty of Medicine, Medical Center, University of Freiburg, Freiburg im Breisgau, Germany

**Keywords:** metabolite, metabolomics, mass spectrometry, MS-imaging, method optimization

The fundamental importance of metabolism is increasingly recognized in many areas of the life sciences (Beger et al., 2016). Not surprisingly, metabolomics, the science of measuring multiple metabolites simultaneously, is a rapidly developing field of research. In the slipstream of more established omics technologies such as transcriptomics and proteomics, metabolomics is in the process of becoming a mainstream technology. The more established omics technologies have shaped the expectations of the broader life science community with respect to standardization, the minimum amount of input material (sometimes a single cell), and the number of features identified from each sample. The articles in this research topic represent important advances in mass spectrometry (MS)-based metabolomics that will enable an even broader application of metabolomics in the future.

Due to the typical complexity of samples, mass resolution alone rarely provides sufficient information. Therefore, mass spectrometry is usually coupled with a second dimension of separation. The most common second dimension is chromatography, which separates analytes by their physicochemical properties (Harrieder et al., 2022). This can reduce differences in matrix effects across samples and aid in compound annotation. In recent years, the spatial distribution of metabolites has gained importance as an alternative dimension of separation (Pade et al., 2023). Two articles in this issue demonstrate such potential and show ways to reduce the impact of matrix effects (Molenaar et al.) and to improve the annotation of detected compounds (Lukowski et al.).

Generally, metabolomics experiments include three main steps: Sample preparation, data acquisition, and data analysis. Two articles in this research topic present an optimization of sample preparation protocols. Henne et al. outline a simplified metabolite extraction method for adherent cultured cells, without cell-scraping, and compatible with multi-well plate protocols for high-throughput GC-MS measurement. The optimized method, which was tested on six different cell types, reduces sample preparation time by about 50% and has a quantitation reliability comparable to standard methods. For lung tissue, Lukowski et al. evaluated agarose inflation to improve preservation of 3D tissue organization without compromising data acquisition by MS imaging. This optimization revealed unique lipid distributions in human lung airway, as well as the spatial co-occurrence with proteins identified *via* high-resolution proteomics of micro-dissected samples. Further in the field of MS imaging, Molenaar et al. show an innovative approach to improve the quantitation of metabolites by introducing fluorescein diacetate as an internal standard that can be detected in fluorescent microscopy and MS alike. As proof of principle, this data-driven compensation of matrix effect at the single cell level improved the separation of co-cultured HeLa and NIH3T3 cells. Looking at MS detection in even more detail, the comprehensive package of a data acquisition method and a matching data processing pipeline presented by Dmitrenko et al. aims to ensure the highest possible data quality while tracking and potentially optimizing ToF-MS hardware performance. Based on a pilot trial of 21 months that included 153 individual measurements of a QC mixture, the study serves as a guide to evaluate system suitability prior to the measurement of biological samples, which considers instrument retuning strategies and preventive maintenance.

Taken together, the articles in this issue tackle highly relevant methodological challenges. This work paves the way for even more widespread application of metabolomics in the life sciences.
